# The Poor Man's Home.—X

**Published:** 1897-10-23

**Authors:** 


### Modern Sociology.

### THE POOR MAN'S HOME.—X.

### The Municipal Landlord—(*continued*).

The London County Council has only lately taken up the question of housing, but it has thus benefited by the experience of other centres, and the work done by it is among the best that Britain can show. It has cleared out a large area in Bethnal Green, and replaced squalid and unhealthy dens by good and sanitary houses. To anyone who knows Bethnal Green it will always be a matter of surprise that so much sympathy was, a few years ago, lavished on Whitechapel. Nowhere in London could you have found more of the signs of poverty, more dreary streets—to honour by this name the foul and winding alleys one traversed—or more dilapidated and visibly insanitary houses than in Bethnal Green. And it was a district so wholly bad that isolated improvements, such as private enterprise can undertake, would have been of little use. The work of the County Council—the Boundary Street scheme, as it is called—has involved the clearing of no less than fifteen acres and the closing of twenty streets. The houses which have been demolished number more than 700. The area thus cleared has been carefully laid out. In the centre of the space is a circular garden, from which seven avenues branch out. These streets average about 50 feet in width—a great change from the narrow lanes of old Bethnal Green. A change as great marks the houses that line the streets. These comprise dwellings of two classes. The best are practically self-contained, each house opening from the corridor or landing, and having all sanitary conveniences within its own door. These dwellings are of different sizes—one, two, or three rooms, the majority being of the larger dimensions. In these the living-room measures 13 feet 7 inches by 11 feet 4 inches. It is provided with a grate, a dresser, and against the outer wall a ventilated food cupboard for food. Pegs for hanging garments are added to the furnishing, and the room is arranged to hold a bed if necessary. At the back of the living-room is a scullery, provided with a sink and a copper. The bedroom in the two-room tenement measures 12 feet 3 inches by 8 feet 6 inches. In the three-room houses there is another bedroom, seven inches longer but a little narrower than the first. The water-closets are entirely separate from the living-rooms, and are well flushed and, as well as the soil-pipes from the sculleries, thoroughly ventilated. The inferior dwellings have living accommodation very similar to these we have described, but for the sake of economy it has been arranged that the sculleries and closets shall be used in common. While this makes the houses less desirable, it permits of their being let at cheaper rents—a point which the prospective tenants are likely to regard as being of more importance; and as the dwellings are meant for the very poor, considerations of privacy had to give way to economy. Even so, it is doubtful if the improvement can be regarded as a remunerative investment except in public health; for the cost of the building has been much greater than a private landlord would care to spend on houses intended for the class of tenants who live in Bethnal Green. This scheme is, indeed, the kind which only a public body will readily undertake, as so much of the gain must come from the bettering of the health and possibly the habits of the occupants. The cost of the scheme has been about a million sterling. By it 5,719 persons have been dispossessed, but as accommodation has been provided for 4,700 it is shown that by good management people can be housed healthily, and yet with fair compactness. To provide healthy accommodation for four-fifths of those who were living under very bad sanitary conditions before on the same space, is no mean feat for an architect.

The Birmingham Corporation also has built a number of dwellings for the working class. As in the case of Glasgow and Liverpool, the Corporation, having cleared a large area, expected private builders to apply for sites, and were disappointed in this expectation. The Improvement Committee therefore obtained the consent of the City Council to their undertaking themselves to build upon it. The houses which they have erected are of a superior class. They are cottages containing four apartments and an attic. Each house has a front and a back door, so that perfect ventilation is attainable. The rents run from 5s. to 6s. 3d. per week. Those at the higher rent have both a living-room and a kitchen (the former 13 ft. square, the latter 12 ft. by 9 ft.), and a pantry behind the kitchen. In the cheaper houses the kitchen is smaller, indeed little more than a scullery. In both there are two bedrooms on the upper storey. Grates are provided, and ovens in the kitchens. Each house has also a closet arranged on good sanitary principles. The Corporation owns 103 cottages of this description, representing a total cost of £14,000.

The Corporation of Huddersfield also is an owner of house property of the cottage type. The 98 houses which it has built are similar in arrangement to those in Birmingham, but they own also some remodelled "back-to-back" houses, which contain only three rooms each. The rent of the new cottages is 4s. 8d. per week, which is a little less than is asked for similar accommodation by private landlords in the town.

At the present time there is a scheme on foot in Dublin by which the Corporation hopes to house a large number of the poorest of the population. The dwellings there also are to include tenements of one, two, and three rooms. The rent for a single room will, it is estimated, be eighteenpence. The usual objections to one-room dwellings have been urged; but, as Sir Charles Cameron says, a large number of the poorest of the poor live in one-room houses as it is, and can afford to live in no other. It is at least better that their single room should be well-built and healthy than that it should be the reverse, as is now too frequently the case.

The commercial fairness of these municipal experiments in landlordism is a difficult question. When, as in Bethnal Green and probably in Dublin, the Corporation or other public body caters for the very poor, those for whom apparently no one else will cater in this essential matter of healthy lodging, we think that no objection can be taken to it. While on general principles it is not good for a public body to enter into competition with private ones, it is usually a good thing when the private builder competes with the public one. One of the best results of civic housing is that it makes the private builder see that he must give good accommodation also. And if he proceeds with ordinary prudence and business-like foresight, he can, as the various semi-philanthropic experiments we have spoken of show, reap a fair remuneration. More we cannot desire him to obtain. Whoever is rackrented, let it not be the poor.

